# The Anti-Proliferative Effects of the CHFR Depend on the Forkhead Associated Domain, but not E3 Ligase Activity Mediated by Ring Finger Domain

**DOI:** 10.1371/journal.pone.0001776

**Published:** 2008-03-12

**Authors:** Tomokazu Fukuda, Yasuyuki Kondo, Hitoshi Nakagama

**Affiliations:** Biochemistry Division, National Cancer Center Research Institute, Tokyo, Japan; University of Arkansas, United States of America

## Abstract

The CHFR protein comprises fork head associated- (FHA) and RING-finger (RF) domain and is frequently downregulated in human colon and gastric cancers up to 50%. The loss of CHFR mRNA expression is a consequence of promoter methylation, suggesting a tumor suppressor role for this gene in gastrointestinal carcinogenesis. In terms of the biological functions of CHFR, it has been shown to activate cell cycle checkpoint when cells are treated with microtubule depolymerizing agents. Furthermore, CHFR was reported to have E3 ligase activity and promote ubiquitination and degradation of oncogenic proteins such as Aurora A and polo-like kinase 1. However, molecular pathways involved in the tumor suppressive function of CHFR are not yet clear since the two established roles of this protein are likely to inhibit cell growth. In this study, we have identified that the FHA domain of CHFR protein is critical for growth suppressive properties, whereas the RF and cysteine rich domains (Cys) are not required for this function. In contrast, the RF and Cys domains are essential for E3 ligase activity of CHFR. By the use of a cell cycle checkpoint assay, we also confirmed that the FHA domain of CHFR plays an important role in initiating a cell cycle arrest at G2/M, indicating a functional link exists between the anti-proliferative effects and checkpoint function of this tumor suppressor protein via this domain. Collectively, our data show that the checkpoint function of the FHA domain of CHFR is a core component of anti-proliferative properties against the gastrointestinal carcinogenesis.

## Introduction

CHFR (Checkpoint protein with Forkhead associated and Ring finger domain) was first isolated by a homology screening of EST cDNA clones harboring an FHA domain [1]. The CHFR protein is characterized by the existence of two domain structures that are well conserved across different species, namely the FHA and RING finger domains (RF) [1]. CHFR is in fact the only protein in vertebrates that contains both of these functional domains.

The FHA domain of CHFR has been reported to arrest the cell cycle under mitotic stress conditions caused by microtubule depolymerizing agents such as nocodazole, and this moiety thus confers a mitotic checkpoint function upon this protein [2]–[6]. In terms of the mechanisms underlying this checkpoint function, CHFR has been shown to exclude Cyclin B1 from the nucleus, resulting in the arrest of the cell cycle at around the G2 phase [7]. Other checkpoint regulators with an FHA domain, such as CHK2 and NBS1, have also shown similar features and arrest the cell cycle in response to DNA damage and replication blocks [8], [9].

These checkpoint proteins containing FHA domain have been shown to function as tumor suppressors, although the detailed molecular mechanisms are not yet fully elucidated. For example, the inactivation of the CHK2 and NBS1 proteins increases the predisposition of cells to cancer development [8], [10]–[14]. The functional inactivation of CHFR due to promoter methylation and the consequent loss of mRNA expression is frequently observed in human colon and gastric cancers [4], [15]–[19], suggesting its possible role also as a tumor suppressor. The functional loss of these checkpoint proteins is likely to disrupt the cell cycle arrest response to cellular stress, thus leading to the accumulation of mutations and replication errors in the genome, a prerequisite for malignant transformation.

The RING-finger domain is a characteristic feature of the E3 ligase proteins [1] and is thought to determine the substrate specificity for ubiquitination reactions. As an example, the RING-finger protein cdc20 is known to serve as an E3 ligase for the anaphase promoting complex/cyclosome (APC/C) [20], and Cyclin B is also one of its substrates [20]. Cyclin B proteins that have been polyubiquitinated by cdc20 are rapidly transferred to the proteasome and degraded. CHFR was shown to play a role as E3 ligase for the polyubiqutination of Aurora A and Polo-like-kinase 1 [21], [22], possibly resulting in the degradation of these proteins. In fact, mouse embryonic fibroblasts (MEF) derived from *Chfr* knockout mice show elevated protein levels of Aurora A and display chromosome abnormalities [21]. The inactivation of CHFR may thus cause the up-regulation of these proteins, which are known mitotic kinases and are frequently observed to be overexpressed in various types of human malignant tumors, such as bladder and colon cancers [23], [24]. Elevated levels of Aurora A and Plk1 are known to induce abnormal mitotic cell division and cause karyotype abnormalities or malignant transformation [25], [26]. The functional loss of CHFR could therefore result in the accumulation of oncogenic proteins (Aurora A and Plk1) and induce genomic instability.

To date, two possible molecular pathways have been considered as the mechanisms underlying the tumor suppressor function of CHFR. These are the checkpoint regulation and E3 ligase functions of this protein. However, it is difficult to draw any conclusions from the findings of previous studies about which of these roles is the most critical for growth suppression, since functional analyses of the checkpoint and E3 ligase activity of CHFR have only been performed independently of each other thus far [1], [22], [27]–[29]. We initially focused on the E3 ligase activity as a possible pathway for the growth suppressive properties of CHFR as our previous data have shown that degradation by the proteasome is the major rate limiting step in the control of the Aurora A protein levels [30]. If the E3 ligase activity of CHFR is more important for growth suppression, as we initially expected, the core region of this tumor suppressor that is required for its anti-proliferative effects was anticipated to be the RF domain.

In our current study, we have investigated the molecular pathways underlying the growth suppressive functions of CHFR by utilizing genetic rescue experiments with colon cancer cell lines in which endogenous CHFR is epigenetically inactivated.

## Materials and Methods

### CHFR plasmids

A cDNA fragment of human CHFR (NIH Mammalian Gene Collection ID: 19963) was obtained by RT-PCR from a human pancreatic cancer cell line (Panc1) based on a method described previously [30]. A hemagglutinin (HA) protein tag sequence was then introduced at the amino terminus of this recombinant CHFR product and a Kozak sequence was inserted just upstream of the start codon of the HA protein tag. Mutant cDNA fragments were created using the quick change PCR kit (Stratagene) with slight modifications [31]. To generate a ΔFHA mutant, a CHFR cDNA fragment was generated that lacked the 110 amino acids between the original start methionine and the end of the FHA domain (MERPEEGKQS-EPEHNVAYLYESLS). An RF mutant (ΔRF) was similarly designed by generating a truncated CHFR cDNA lacking this 48 amino acid domain (CIICQDLLHD-TCRCPVERICK). A TGA stop codon was inserted into the CHFR cDNA to construct the cysteine rich domain mutant (ΔCys) by producing a protein product lacking the 190 amino acids of this region (VCPLQGSHAL-HICEQTRFKN).

Each cDNA was subcloned into the EcoRV site of pBluescript SKII+ (Stratagene) by blunt end ligation, and the resulting constructs were validated in a cycle sequencing reaction with an ABI 310 genetic analyzer (Applied Biosystems). Both the wild-type and mutant CHFR cDNA fragments were also subcloned into an LXIN retrovirus vector (Clontech), the pcDNA 3.1+ plasmid (Invitrogen), and an IRES2-EGFP bicistronic expression vector (Clontech). The retrovirus vectors were used in colony formation assays, whereas the pcDNA 3.1+ vectors were used in an *in vivo* ubiquitination assay and also in the cellular localization experiments. The bicistronic vectors (IRES2-EGFP) were employed in the checkpoint analyses.

### Stable and transient expression of wild-type and mutant CHFR proteins in cultured cells

HCT116, RKO and HeLa cells were cultured in Dulbecco's Modified Eagle's Medium (DMEM; Sigma) supplemented with 10% fetal bovine serum (FBS), penicillin (100U/ml), and streptomycin (50U/ml), at 37°C and 5% CO_2_. A PT67 retrovirus packaging cell line was obtained from Clontech, and maintained in DMEM with 10% FBS. Cells were maintained in an exponential growth phase prior to use.

Retroviral constructs harboring wild-type or mutant human *CHFR* cDNAs, were introduced into PT67 cells (Clontech) for packaging using the lipofection method (Fugene 6, Roche). The LXIN retroviral vector contains a neomycin cassette, and cells that stably produced recombinant retroviruses were selected after two weeks of culture in the presence of 1 mg/ml G418. The retroviral supernatants were diluted 1:2 with normal DMEM containing 10% FBS and exposed to HCT116 or RKO cells with 400 µg/ml of polybrane infection enhancer. Infected cells were further selected with 1 mg/ml G418 for two weeks. To avoid cloning bias, the whole cell population that showed resistance to G418 was used in each experiment. For transient expression experiments, cells at 70% confluency were transfected with 2.5 µg of the indicated plasmids using the lipofection method (Lipofectamine 2000, Invitrogen), according to the manufacturer's protocol.

### Colony formation assay

1×10^6^ HCT116, RKO or HeLa cells were infected with aliquots of LXIN retroviral supernatants from the PT67 packaging cells for 48 h. When the infected cells reached confluence, they were trypsinized and resuspended in 10 ml of DMEM supplemented with 10% FBS. 10 µl (HCT116) or 100 µl (RKO or HeLa) aliquots of these cell suspensions were then seeded into 100 mm culture dishes (Nunc, 150350), and grown in G418 selection for two weeks as described above. G418-resistant colonies were subsequently fixed in 4% paraformaldehyde in phosphate buffered saline (PBS), and stained with hematoxilin. Images of the stained dishes were captured using a high-resolution digital camera with a macro lens (Fuji Film), and the numbers of colonies were determined in each image using NIH image software. The average colony numbers were then calculated from five dishes in three independent experiments. Statistical significance was evaluated with the Wilcoxon-Mann-Whitney *U*-test.

### Measurement of the retrovirus titers of the producer cells

The conditioned medium of the producer cells was diluted 1:50 with distilled water and subjected to reverse transcription. The copy number of the resulting retroviral cDNA was measured by real-time PCR with a Retrovirus titer set (Takara Bio, Kyoto, Japan). The average retrovirus copy number of per ml was determined from five independent reactions. The reverse transcriptase and real-time PCR reactions were performed according to the manufacturer's instructions.

### Western blot analysis

Cells were lysed in ice-cold HIPS buffer (50 mM Tris-HCl, pH 7.5, 150 mM NaCl, 1% Triton X-100) [30] and the resulting whole cell lysates were subjected to 10% SDS-PAGE. The separated proteins were then transferred to polyvinylidene difluoride (PVDF) membranes (Immobilon P, Millipore). After blocking with 7% non-fat dry milk-Tris buffered saline and 0.1% Tween 20 (TBST), the membranes were probed with anti-HA (High affinity HA 3F10, 1/5,000 dilution, Roche), and anti-α-tubulin (DM-1A, 1/5,000 dilution, ICN Biomedicals) antibodies. Blots were then incubated with horseradish peroxidase (HRP)-conjugated rabbit anti-rat IgG (A5795, 1/5,000 dilution, Roche) or donkey anti-mouse IgG (NA1093V, 1/5,000 dilution, GE Healthcare Bioscience) secondary antibodies respectively, and immunoreactive proteins were detected by enhanced chemiluminescence (P90720, Millipore).

### In vivo ubiquitination assay

2.5 µg of wild-type and mutant CHFR pcDNA 3.1+ expression plasmids, and an empty vector control, were introduced into HCT116 cells at 70% confluency using the lipofection method (Lipofectamine 2000, Invitrogen). 2.5 µg of pcDNA 3.1+ vector harboring FLAG tagged ubiquitin (kindly provided by Dr. K. Miyazono, Tokyo University) was co-transfected with these constructs. After 20 h, the cells were treated with 25 µM of MG132 (C2211, SIGMA) for 5 h, lysed in HIPS buffer, and subjected to immunoprecipitation. A 500 µg aliquot of total protein from the transfected HCT116 cells in 250 µl lysis solution was mixed with 10 µl of the anti-HA affinity matrix (Roche) pre-blocked with 2% bovine serum albumin (BSA), and incubated for 4 h with gentle rotation at 4°C. The affinity matrix was washed with HIPS buffer three times, collected by centrifugation, and the precipitated proteins were denatured in sample buffer containing 0.1M dithiothreitol (DTT), subjected to 7% SDS-PAGE and transferred to a PVDF membrane. The transferred proteins were then incubated with anti-HA (high affinity HA 3F10, 1/5,000 dilution, Roche), or anti-FLAG (monoclonal anti-FLAG M2, 1/5,000 dilution, Sigma) antibodies. The secondary antibodies used were as described above for the western blotting procedure.

### Intracellular localization of CHFR protein

pcDNA3.1+ expression vectors were introduced into HCT116 cells using the lipofection method, as described earlier. Twenty-four hours after transfection, the cells were fixed with 4% paraformaldehyde and then treated with 0.5% Triton X-100 (both in PBS) for 5 min. The cells were preblocked with 1% normal goat serum (NGS) in PBS for 30 min and incubated with an anti-HA antibody (high affinity HA 3F10, 1∶500 dilution, Roche) for 1 h at room temperature. An alexa 488-conjugated goat anti-rat IgG (Invitrogen) was used as the secondary antibody. A rabbit PML antibody (PM001, 1/300 dilution, MBL) and alexa 594-conjugated goat anti-rabbit IgG (Invitrogen) were used to detect PML nuclear foci. The cells were counter-stained in each case with 1μM of Hoechst 33258 (B1155, Sigma) and staining images were captured using an Axiovart (Zeiss) fluorescence microscope.

### Cell cycle checkpoint analysis

For checkpoint analysis, HCT116 cells were transfected with IRES2-EGFP expression plasmids harboring wild-type or mutant CHFR inserts for 5 h and then treated with nocodazole at a concentration of 200 ng/ml (from a 100 mg/ml stock solution in dimethyl sulfoxide, Sigma) for 16 h. After the nocodazole treatment, the cells were fixed in 4% paraformaldehyde/PBS and the numbers of mitotic cells, which show EGFP fluorescence, were determined by microscopic examination. The average percentage of the total EGFP-positive cells that were deemed to be mitotic was calculated by counting approximately 100 cells expressing either wild-type or mutant CHFR proteins.

## Results

### The expression of wild-type and mutant CHFR proteins in colon cancer cells which lack the endogenous species

**Figure 1:**
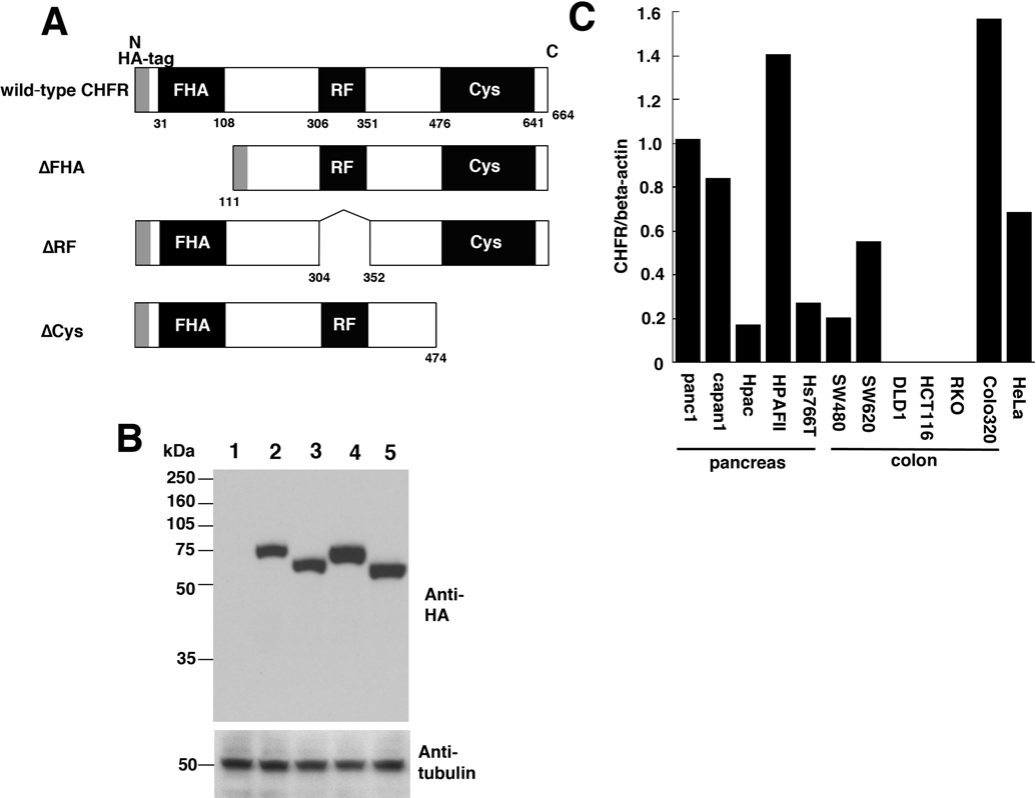
Schematic representation of the wild-type, ΔFHA, ΔRF and ΔCys forms of the CHFR protein tagged with HA, and expression analysis of these proteins in a colon cancer-derived cell line that lacks endogenous CHFR mRNA. (A) The predicted protein structures of the wild-type, ΔFHA, ΔRF and ΔCys CHFR proteins. N and C indicate the amino and carboxyl terminus, respectively. FHA, forkhead associated domain; RF, ring finger domain; Cys, cysteine rich domain; HA-tag, hemagglutinin protein tag. (B) The detection of the wild-type, ΔFHA, ΔRF and ΔCys forms of CHFR proteins transiently expressed in HCT116 cells by western blotting with anti-HA antibodies (upper panel). Cells were transfected with HA-tagged empty vector (lane 1); wild-type CHFR (lane 2); ΔFHA (lane 3); ΔRF (lane 4); or ΔCys (lane 5). Tubulin was also detected as a loading control (lower panel). (C) Endogenous CHFR mRNA expression levels detected by real-time PCR in the indicated pancreatic- and colon cancer-derived cell lines. The relative levels of CHFR mRNA shown are normalized to beta-actin mRNA, the expression level of panc1 was set as 1.0, and the average values of duplicate experiments were calculated.

Colon cancer cells have lost the endogenous expression of CHFR, and we thus anticipated that this would be an appropriate cell system to elucidate the functional domain responsible for the anti-proliferative effects of CHFR protein by genetic rescue. The endogenous levels of *CHFR* mRNA were measured by real-time RT-PCR in five pancreatic cancer cell lines and six colon cancer cell lines (Fig. 1C). *CHFR* transcripts were detectable in each of the pancreas cell lines tested but three colon cancer derived cell lines, HCT116, RKO and DLD1, showed no expression of *CHFR* mRNA.

The structures of the wild-type and mutant CHFR proteins are shown in Figure 1A. Expression vectors for these proteins were introduced transiently into HCT116 cells and subjected to western blotting with anti-HA antibodies to determine whether the predicted recombinant CHFR proteins were produced (Fig. 1B). Positive bands were indeed detected at the expected molecular weights (wild-type; 72 kDa, ΔFHA; 57 kDa, ΔRF; 66 kDa, ΔCys; 50 kDa), indicating that each CHFR protein product was efficiently expressed.

### The anti-proliferative effects of CHFR are dependent upon the FHA domain and not the RF or Cys domains

**Figure 2:**
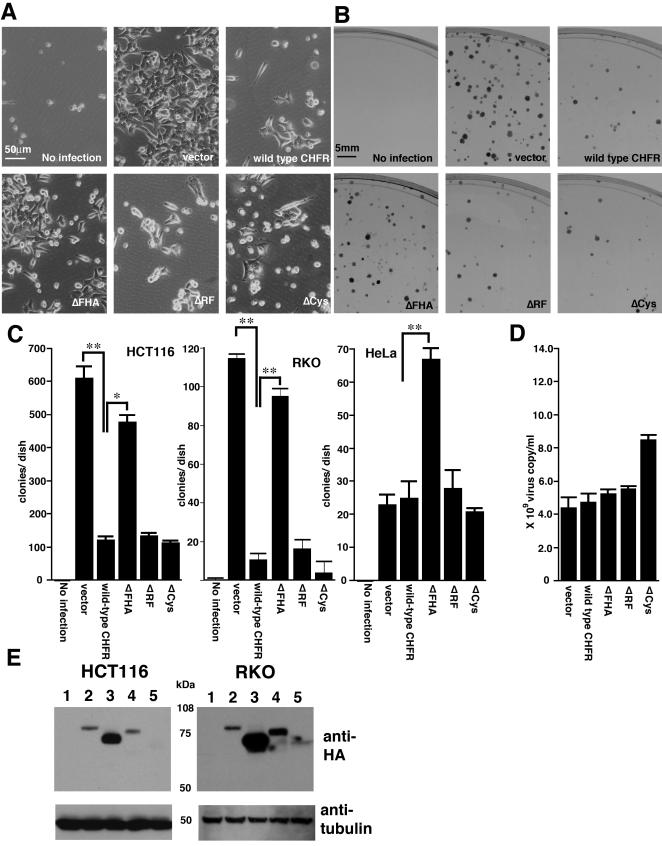
Identification of the functional domain of the CHFR protein that confers its anti-proliferative effects. (A) The growth appearance of HCT116 cells infected with retroviral vectors expressing the indicated CHFR products. Note that the wild-type CHFR, ΔRF and ΔCys retroviruses suppressed the cell growth of the host cells and this was partially restored in the ΔFHA expressing cells. There were no differences, however, between any of these transfected cells in terms of their morphology. (B) Colony formation assay of HCT116 cells after retroviral infection with the same CHFR constructs as in (A). (C) Statistical evaluation of the colony formation assay results for HCT116, RKO and HeLa cells expressing the indicated exogenous proteins. *p<0.01, **p<0.05. (D) Measurements of the recombinant retrovirus copy number in the supernatants of PT67 producer cells by real time PCR. (E) The detection of stably expressed wild-type, ΔFHA, a ΔRF and ΔCys CHFR protein in HCT116 and RKO cells following infection with the corresponding recombinant retroviruses. Total cellular protein extracts were obtained at the fourth passage. The level of introduced proteins in the cells that infected with empty vector (lane 1); wild-type CHFR (lane 2); ΔFHA (lane 3); ΔRF (lane 4); or ΔCys (lane 5).

To evaluate the effects of wild-type and mutant CHFR proteins (ΔFHA, ΔRF and ΔCys) on cell proliferation, recombinant retroviruses harboring the corresponding cDNAs were introduced into HCT116 cells. There were no major differences found in the resulting cell morphologies in each case, as shown in Figure 2A. Substantial growth suppression was observed following the introduction of the wild-type CHFR during G418 selection (Fig. 2A). In addition, whereas the ΔRF and ΔCys mutants showed growth suppressive effects that were almost identical to the wild-type CHFR, the ΔFHA protein had minimal inhibitory effects upon cell proliferation (Fig. 2A).

To more quantitatively evaluate the growth inhibitory effects of the wild-type and mutant CHFR proteins, a colony formation assay was carried out using the corresponding retrovirally infected HCT116 cells under G418 selection (Fig. 2B and C). The expression of wild-type, ΔRF and ΔCys proteins resulted in a significant decrease in the number of G418-resistant colonies in this experiment compared with the vector control (p<0.01), whereas ΔFHA expressing cells did not show any reduction in colony number. The same recombinant *CHFR* retroviruses were also introduced into RKO cells and similar results were observed (Fig. 2C).

To verify the titer of our recombinant CHFR retroviruses, the virus copy numbers in the conditioned supernatant of the PT67 producer cells were measured by real-time PCR. As shown in Figure 2D, no major differences could be observed in the retrovirus copy number between the wild-type and mutant CHFR retrovirus producer cells. We therefore concluded that the growth suppressive effects of CHFR that we observed in these analyses are not due to any differences in the titers of the producer cells.

### Detection of wild-type and mutant CHFR proteins under stable expression conditions

During their initial passages, significant growth suppression was observed in HCT116 cells expressing exogenous wild-type, ΔRF and ΔCys CHFR, as shown in Figures 2A–C. However, this growth suppression becomes almost undetectable by passage 3. The protein levels of each of these introduced CHFR products were thus analyzed in these cells at passage 4 by western blotting. As shown in Figure 2E, the levels of the wild-type, ΔRF and ΔCys products were remarkably low when compared with ΔFHA (Fig. 2E, left panel). The same analysis was undertaken in RKO cells and produced essentially identical results (Fig. 2E, right panel). A possible explanation for this phenomenon is that elevated levels of wild-type, ΔRF and ΔCys proteins may cause a substantial growth disadvantage, and thus cell populations which express these introduced proteins at low levels undergo positive selection with passage in culture.

### The E3 ligase activity of CHFR requires the RF and Cys domains, but not the FHA domain

**Figure 3:**
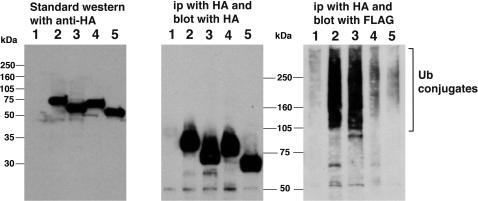
* In vivo* ubiquitination assay for the wild-type, ΔFHA, ΔRF and ΔCys forms of the CHFR protein. (Left panel) Measurement of the transient expression levels of both the wild-type and mutant form of CHFR proteins in HCT116 cells by western blotting. The proteins were separated by 10% SDS-PAGE. (Middle panel) Immunoprecipitation and immunoblotting of proteins with anti-HA antibodies. The proteins were resolved by 7.5% SDS-PAGE. (Right panel) The detection of FLAG-reactive proteins following immunoprecipitation by HA antibodies. Ubiquitinated proteins are evident by the presence of high molecular weight bands in lanes 2 and 3, but not in lanes 1, 4 and 5. Cells were cotransfected with FLAG tagged ubiquitin expression (FLAG-Ubi) vector and either empty vector (lane 1); wild-type CHFR (lane 2); ΔFHA (lane 3); ΔRF (lane 4); or ΔCys (lane 5).

A series of *in vivo* ubiquitination assays were conducted using HCT116 cell populations that transiently expressed the wild-type and mutant CHFR proteins at similar levels (Fig 3, left panel). The recombinant CHFR and its substrate proteins were immunoprecipitated from the corresponding cellular extracts with an anti-HA antibody and the ubiquitination activity levels were then monitored by western analysis with anti-FLAG antibodies to detect high molecular weight bands as described in a previous study [32]. As shown in the right panel of Figure 3, polyubiquitinated proteins were detectable in cells expressing wild-type and ΔFHA proteins but not the ΔRF and ΔCys mutants, which is deemed to be substrates of CHFR. The mobilities of these immunoprecipitated CHFR proteins were also not altered as a result of polyubiquitination (Fig. 3, middle panel), indicating that no self-ubiquitination had occurred in each case. From these results, we concluded that E3 ligase activity of CHFR requires the RF and Cys domains, but is unaffected by the deletion of the FHA domain.

### Analysis of the intracellular localization of wild type, ΔFHA, ΔRF and ΔCys CHFR proteins by immunofluorescence staining

**Figure 4:**
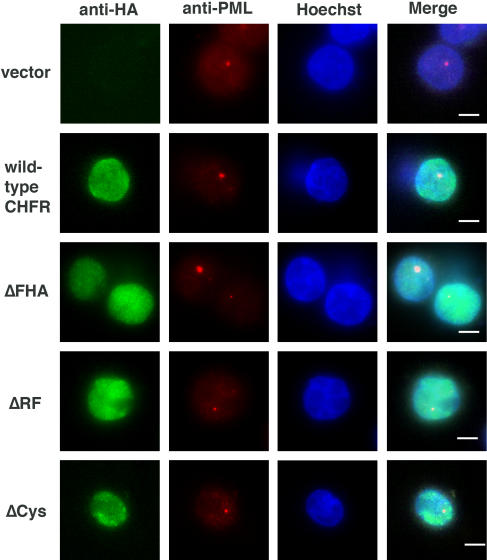
Intracellular localization of wild-type and mutant forms of CHFR proteins detected by fluorescence immunostaining. The localization of the wild-type or mutant forms of the CHFR protein was assessed by the immunoreactivity of an anti-HA antibody (green signal). PML nuclear foci were detected with an anti-PML antibody (red signal). The nuclei of HCT116 cells were counterstained with Hoechst (blue). Merged pictures are shown to highlight the signal overlap between the Hoechst and anti-HA staining.

A previous study has indicated that the FHA domain of the CHFR protein is essential for its localization in promyelocytic leukemia (PML) foci within the nucleus [33]. The intracellular localization of the wild type and mutant CHFR proteins was detected by immunostaining of HCT116 cells that exogenously expressed these products. As shown in Figure 4, each of these introduced CHFR species showed a diffuse nuclear localization and no differences could be observed. Although the cells were doubly stained with anti-HA and anti-PML antibodies, a dominant accumulation of CHFR within the PML nuclear foci was not evident for either the wild type or mutant proteins. From these data, we conclude that the CHFR domains analyzed do not impact upon the intracellular localization of this protein.

### The checkpoint function of CHFR requires the FHA domain only

**Figure 5:**
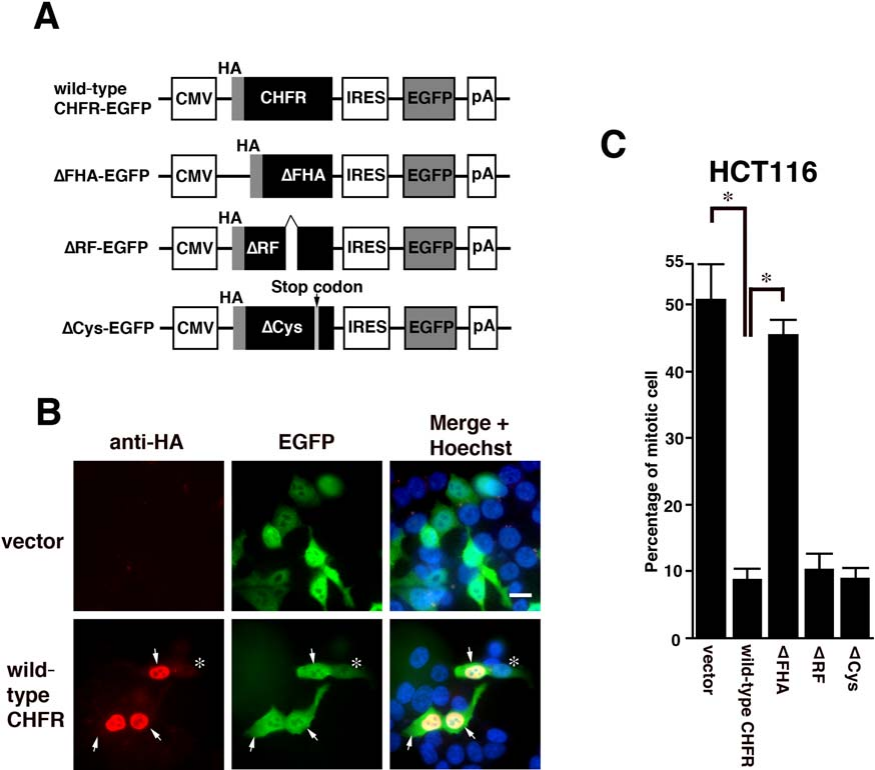
Checkpoint assay of the wild type and mutant forms of the CHFR protein. (A) Schematic representation of the bicistronic vectors used to express the indicated CHFR products. (B) Validation of the positive correlation between the anti-HA antibody immunoreactivity and EGFP expression profiles. Cells that were positive for both are denoted by arrows. Cells that were positive for the anti-HA antibody, but not for EGFP, are highlighted by an asterisk. (C) Mitotic checkpoint analysis of HCT116 cells after the introduction of an empty vector, wild-type-EGFP, ΔFHA-EGFP, ΔRF-EGFP or ΔCys-EGFP. Asterisks indicate statistical significance (p<0.05).

The functional recovery of the checkpoint function of CHFR was evaluated also in HCT116 cells after the exogenous introduction of wild type and mutant CHFR proteins. Since the transfection efficiency was limited to around 5% for the transient expression vector, a bicistronic expression vector harboring the CHFR wild type and mutant inserts was used in these experiments (Fig. 5A). Since in this case the CHFR and EGFP protein products are translated from the same mRNA, EGFP fluorescence can be used as a marker of recombinant CHFR protein expression. To validate the positive correlation between EGFP expression and CHFR expression, the percentage of EGFP positive cells that were immunoreactive also for the anti-HA antibody was determined microscopically (Fig 5B). Among the 200 EGFP-positive cells that were counted, 182 (91%) showed positive staining for anti-HA antibody (Fig. 5B), confirming the usefulness of EGFP as a marker. As shown in Figure 5C, following nocodazole treatment, the percentage of EGFP-positive mitotic cells was found to decrease in conjunction with the expression of the wild type, ΔRF and ΔCys CHFR proteins, compared with the vector control. In contrast, the expression of the ΔFHA mutant did not affect the percentage of mitotic cells. From these data, we conclude that the FHA domain is essential for the mitotic checkpoint function of CHFR.

## Discussion

Our current study has identified the CHFR domains that are responsible for the anti-proliferative effects of this protein and the results are summarized in Table 1. We find that both the anti-proliferative effects and checkpoint function of CHFR require the FHA domain, whereas the E3 ligase activity of this tumor suppressor relies on the RF and Cys domains. This suggests that there is a functional link between the anti-proliferative effects and checkpoint function of CHFR. Our present analyses also reveal that the FHA domain is the most important region of CHFR for its anti-proliferative role, which is in good agreement with several previous studies. Toyota *et al* have also reported that the introduction of a wild-type CHFR expression vector causes the growth suppression of the host cells, whereas a FHA deletion mutant did not affect cell growth [19].

**Table 1:** Summary of the functional properties of wild-type, ΔFHA, ΔRF and ΔCys CHFR proteins.

	Wild type CHFR	ΔFHA	ΔRF	ΔCys
Anti-proliferative effects	Yes	No	Yes	Yes
E3 ligase activity	Yes	Yes	No	No
Cellular localization	Nucleus	Nucleus	Nucleus	Nucleus
Checkpoint function	Yes	No	Yes	Yes

Although the data presented by Toyota *et al* was quite suggestive of the essential role of FHA in cell growth control, it should be noted that the cell lines they analyzed (SW480 and T98G) endogenously express CHFR transcripts (see Fig. 1C for SW480) [34]. The accurate and quantitative evaluation of the impact of the ΔFHA mutant on cell growth is difficult to undertake in the presence of endogenous protein because of the dominant-negative properties of this mutant [1]. Interestingly, we detected that the expression of these mutant CHFR proteins had a completely different result upon the growth of HeLa cells, which express endogenous CHFR, compared with HCT116 and RKO cells (Fig. 2C). In these experiments, the expression of ΔFHA induced a significant increase in colony number whereas the wild-type, ΔRF and ΔCys CHFR species did not show any effects (Fig. 2C, Right panel). A previous study has indicated that the ΔFHA mutant of CHFR acts as a dominant-negative and can disrupt the checkpoint function of the wild type protein [1]. Hence, we speculated that the increase in the colony number in HeLa cells by ΔFHA was caused by the dominant-negative effects of this mutant upon the endogenous wild type CHFR protein [1]. The reported existence of a splicing variant form of CHFR that lacks the FHA domain and acts in a dominant-negative manner further supports our contention [19]. It has not yet been reported until this manuscript to evaluate the effect of FHA domain against cell proliferation under the two conditions, the existence and non-existence of endogenous CHFR. We therefore conclude that our current study is the first report that precisely identifies the functional domain responsible for the anti-proliferative function of CHFR.

We show from our analysis that the loss of the RF domain and its associated E3 ligase activity did not disrupt the anti-proliferative effects of CHFR, suggesting that the E3 ligase activity of this protein is not essential for its tumor suppressor function. From previous functional analysis of CHFR knockout mice, however, Yu *et al* concluded that the tumor suppressor function of CHFR is conferred by its E3 ligase activity toward the Aurora A protein, a predicted oncogenic kinase [21]. There is therefore some disagreement regarding the precise role of the E3 ligase activity of CHFR, but possible explanation can be considered. We need to pay attention to the tissue specificity of CHFR to induce the degradation of Aurora A. Elevated protein levels of Aurora A are frequently observed in both human colon and mammary cancers [25], [26], but the inactivation of CHFR is limited to cases of colon cancer [17], [35]. High levels of Aurora A in mammary cancers cannot therefore be explained by a loss of function of CHFR. These findings indicate the existence of degradation pathways for Aurora A that are independent of CHFR [17]. In this regard, Cdh1 is a strong candidate as an alternative E3 ligase for Aurora A [36], [37]. The exogenous introduction of a dominant-negative form of Cdh1 was reported to induce elevated levels of Aurora A protein in HeLa cells [37]. Hence, there may be several molecular pathways that promote the degradation of Aurora A but that are tissue- or organ-specific. The protein levels of Aurora A did not change after the expression of any form of CHFR in HCT116 colon cancer cells in our current experiments (data not shown). The differences between the findings of this and other studies might thus be due to the cell-type specificity of the degradation pathways for Aurora A.

With regards to the E3 ligase activity of CHFR, we found that the RF and Cys domains are essential. These results are consistent with previously reported findings, which show that CHFR binds to its substrates through its Cys domain, and forms a protein complex with APC/C through its RF domain [21]. Some E3 ligases have also been reported to have self-ubiquitination activity [38], [39], but we did not detect this in the case of CHFR. In contrast, Kang *et al* have reported strong self-ubiquitination activity for CHFR [22]. One possibility to be taken into consideration when evaluating these discrepancies is the difference of their GST protein tag and our HA tag. The self ubiquitination activity affected by the protein tag has reported with MBP protein tag in case of Rma1, EL5 and Perkin [40]–[42]. Therefore, further experiments with other protein tags would be needed to get the conclusion about the self ubiquitination activity of CHFR protein.

The results of our current study also show that the expression of CHFR arrests the cell cycle prior to entry into mitosis in the presence of microtubule depolymerizing reagents, which is consistent with previous reports [1], [19], [22], [27], [28]. A growth advantage will be conferred upon cells which have lost the checkpoint machinery that functions under mitotic stress conditions. In agreement with this idea, shRNA knock down of CHFR recently reported to induce the increased cell proliferation during the preparation of this manuscript [43]. Since the promoter methylation of the *CHFR* gene was detected in non-invasive adenoma lesions of colon epithelia [44], the associated loss of the checkpoint function of CHFR in these cells may have a significant impact upon the early stages of colon carcinogenesis. To gain further supportive evidence that the FHA domain of CHFR is important for its growth inhibition properties, we have recently put a considerable amount of effort into establishing a conditional expression system for this protein using the Cre-loxP recombination system [30] to evaluate tumor formation activity *in vivo* (e.g. nude mice). Unfortunately, we have yet to establish an efficient conditional system due to the toxicity of Cre-expressing adenovirus in HCT116 colon cancer cells. Although it is not direct evidence, a remarkably high protein level of the ΔFHA mutant, compared with that of the wild-type and other mutant forms of CHFR, suggests that the FHA domain has an important role in suppressing tumor formation (Fig. 2E). The FHA domain is expected to work as the binding motif for phosphorylated proteins. We are currently working on the experiments to isolate the binding partner of FHA domain of CHFR protein. There is a possibility that the detail of anti-proliferative effect can be explained with the binding of new candidates and CHFR. These information will help us to gain further insights in understanding how a functional loss of this gene leads to increased cell proliferation during gastrointestinal carcinogenesis.
